# SIRT3 and SIRT4 are mitochondrial tumor suppressor proteins that connect mitochondrial metabolism and carcinogenesis

**DOI:** 10.1186/2049-3002-2-15

**Published:** 2014-10-20

**Authors:** Yueming Zhu, Yufan Yan, Daniel R Principe, Xianghui Zou, Athanassios Vassilopoulos, David Gius

**Affiliations:** 1 Department of Radiation Oncology, Robert H. Lurie Comprehensive Cancer Center, Feinberg School of Medicine, Northwestern University, Chicago, IL 60611, USA; 2 Department of Surgery, Robert H. Lurie Comprehensive Cancer Center, Feinberg School of Medicine, Northwestern University, Chicago, IL 60611, USA; 3 Department of Radiation Oncology, Northwestern University Feinberg School of Medicine, Rm 3-119, Lurie Research Bldg., 303 East Superior, Chicago, IL 60611, USA

**Keywords:** SIRT3, SIRT4, Acetylome, Acetylation, Carcinogenesis

## Abstract

It is a well-established scientific observation that mammalian cells contain fidelity proteins that appear to protect against and adapt to various forms of endogenous and exogenous cellular conditions. Loss of function or genetic mutation of these fidelity proteins has also been shown to create a cellular environment that is permissive for the development of tumors, suggesting that these proteins also function as tumor suppressors (TSs). While the first identified TSs were confined to either the nucleus and/or the cytoplasm, it seemed logical to hypothesize that the mitochondria may also contain fidelity proteins that serve as TSs. In this regard, it now appears clear that at least two mitochondrial sirtuins function as sensing, watchdog, or TS proteins *in vitro*, *in vivo*, and in human tumor samples. In addition, these new results demonstrate that the mitochondrial anti-aging or fidelity/sensing proteins, SIRT3 and SIRT4, respond to changes in cellular nutrient status to alter the enzymatic activity of specific downstream targets to maintain energy production that matches energy availability and ATP consumption. As such, it is proposed that loss of function or genetic deletion of these mitochondrial genes results in a mismatch of mitochondrial energy metabolism, culminating in a cell phenotype permissive for transformation and tumorigenesis. In addition, these findings clearly suggest that loss of proper mitochondrial metabolism, via loss of SIRT3 and SIRT4, is sufficient to promote carcinogenesis.

## Review

Mammalian cells express proteins that protect against endogenous and exogenous forms of cellular damage to both monitor and maintain the integrity of a cell [1-3]. An extension of this observation would be that the loss of function or genetic mutation of these genes creates a cellular environment that is permissive for the development and/or accumulation of cellular damage that can put the cell at a significantly increased risk for several human illnesses, including cancer [4-7]. Since it is unlikely that evolutionary pressure selected for proteins in mammalian cells to prevent carcinogenesis, these proteins are more likely fidelity proteins that have evolved over time to protect specific organelles from damage by agents that induce genotoxic stress [8]. These proteins are often referred to as tumor suppressors (TSs) as mice lacking these genes tend to develop tumors, and in many cases, these TS genes are deleted or mutated in human tumors [9,10]. While the first identified TSs were confined to either the nucleus and/or the cytoplasm, it seems logical to hypothesize that the mitochondria would also contain fidelity proteins that would serve as TSs.

It now seems clear that the cellular processes that govern or oversee aging, perhaps better defined as longevity, are directed by a combination of complex genetic, biochemical, and cellular pathways that appear to be regulated, at least in part, by a relatively new gene family referred to as sirtuins [11,12]. The sirtuin family genes are the human and murine homologs of the *Saccharomyces cerevisiae Sir2* that have been shown to directly regulate both replicative and overall lifespan [13,14] as well as longevity in *Caenorhabditis elegans* and *D. melanogaster*[11,12]. In these more primitive species, it appears that these genes direct longevity, at least in some significant part, by silencing telomeres and sub-telomeric regions, silent mating type loci, and, crucially, the rDNA, suppressing formation of rDNA circles [12,15].

**Figure 1:**
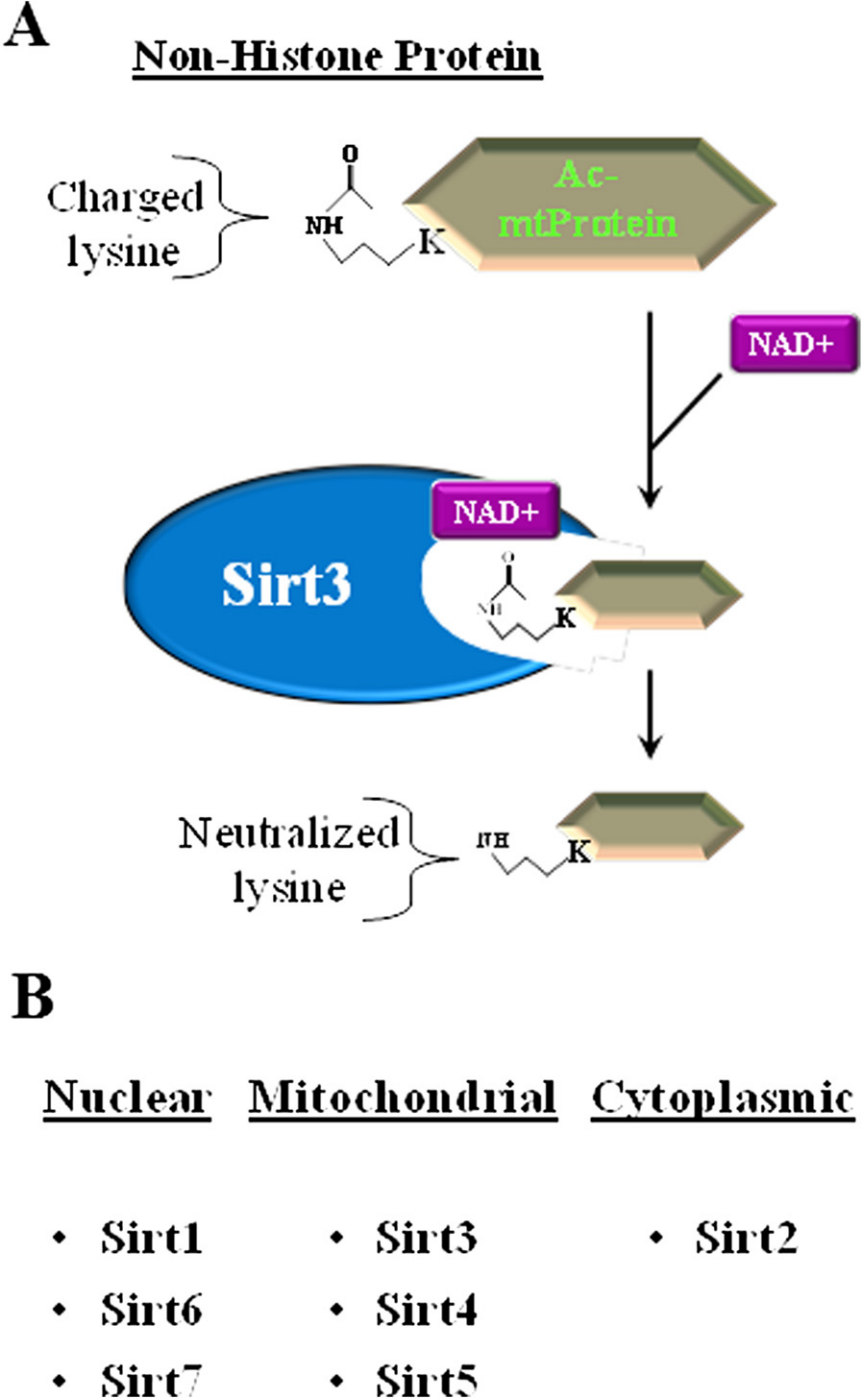
**Overview of sirtuin biology. (A)** Cellular localization of the nuclear, cytoplasmic, and mitochondrial sirtuins. **(B)** Schematic of the enzymatic function of mitochondrial sirtuins using SIRT3.

In contrast, mammalian sirtuin members are associated with numerous physiological roles including stress response, regulation of metabolism, gene silencing, and aging [16,17]. While it has not been shown that these genes determine longevity in mammals, they do appear to regulate critical signaling networks, and following stress, several mice lacking one of the sirtuin genes develop illnesses that mimic those observed in older humans [12,18]. Sirtuins were initially discovered to function as deacetyltransferases; however, it now appears that several of the proteins also function as ribosyltransferases [19-21] under specific conditions. Based on these results, it has been proposed that the mammalian sirtuins play a significant role, at least in part, in directing the acetylome signaling network that has recently been shown to be critical in regulating multiple cellular processes [22]. While this represents only a part of the overall role of sirtuins in mammalian biology, it now appears clear that this protein family, in some significant way, directs the activity of downstream targets via post-translational modifications involving protein acetylation (Figure 1A) to maintain cellular metabolic homeostasis [23].

Mammalian sirtuins are classified as class III histone deacetylases, which are different than traditional class I and II histone deacetylases (HDACs) [24,25]. Unlike conventional HDACs, sirtuins have a variety of non-histone substrates ranging from metabolic enzymes to structural proteins as well as histones [15,19]. The function of sirtuins is very well conserved via a common 275-amino acid catalytic domain, and these proteins are localized to the nucleus (SIRT1, 6, and 7), mitochondria (SIRT3, 4, and 5), and cytoplasm (SIRT2) (Figure 1B) [13]. Sirtuins are NAD^+^-dependent deacetylases, and early on in the investigation of sirtuin biology, it was assumed that their requirement for NAD^+^ implied that their mechanistic activity was connected to cellular metabolism, providing a link between sirtuin activity, energy, and stress responses [26,27]. For instance, the mitochondrial sirtuins, SIRT3 and SIRT4, appear to respond to changes in cellular and nutrient stress, resulting in the activation of their deacetylase or ribosyltransferase activity and hence in post-translational modifications of downstream target proteins [27-31]. It is now well established that SIRT3 deacetylation activity is activated by caloric restriction (CR) and fasting [29-31], and this induction of deacetylation activity also appears to protect against the development of age-related human pathology, including tumorigenesis [29,32]. While these results do not, *a priori*, directly connect sirtuins to longevity, they do strongly suggest that sirtuins, including mitochondrial sirtuins, play a role, at least in some significant part, in the complex process of aging.

### Inflection points, aging, and human carcinogenesis

**Figure 2:**
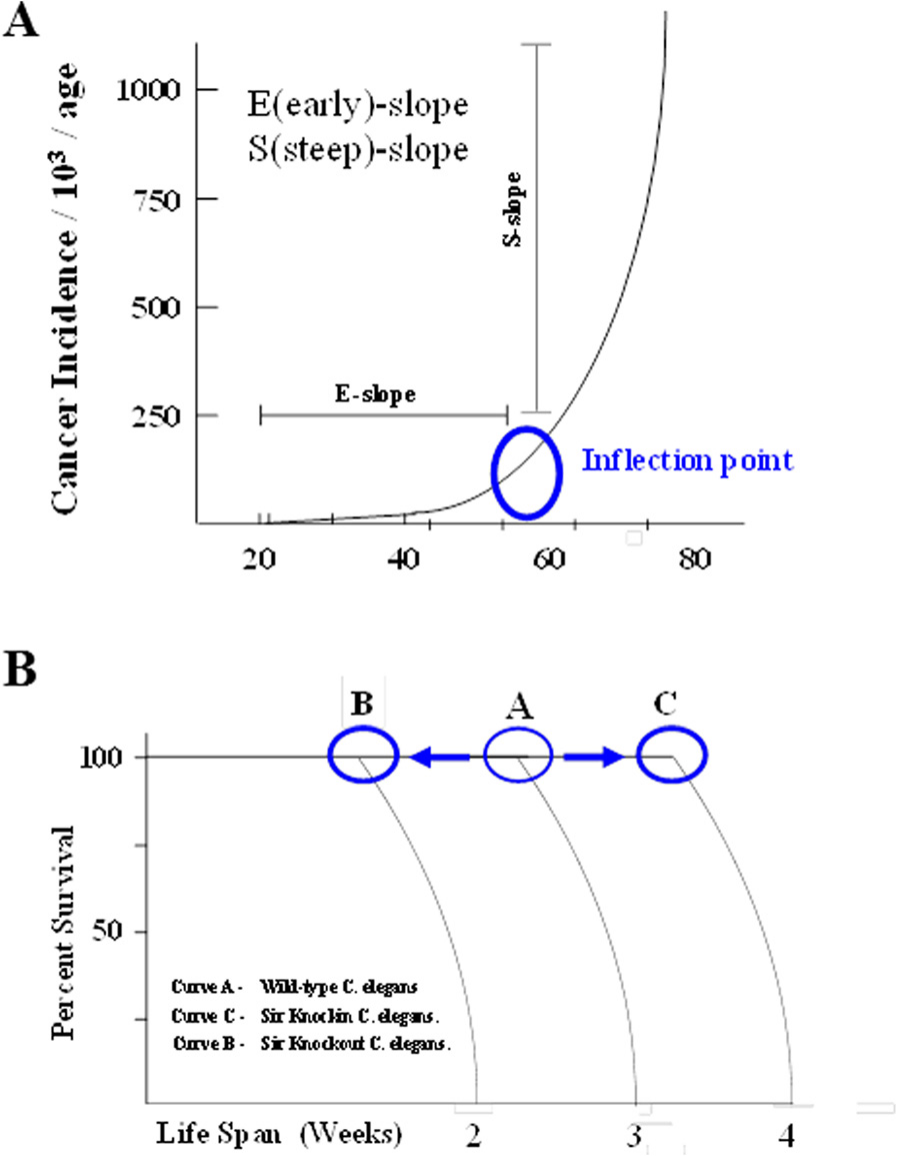
**Cancer incidence rises with age. (A)** The incidence of solid tumor cancers derived from somatic cells increases exponentially with age. The circle marks the inflection point at the transition between the early (E) slope and steep (S) slope. **(B)** The effect of sirtuin gene expression on lifespan. This is a graphical summary of data obtained from increased or decreased sirtuin expression in *C. elegans*. Overexpression of sirtuin genes results in increased lifespan (curve C), whereas underexpression of these genes shortened lifespan (curve B). The time of the inflection point (circle) is shifted, but the general shape of the survival curve remains unchanged.

One fundamental observation in cancer etiology is that the rate of malignancies in any mammalian population increases exponentially as a function of age, suggesting a mechanistic link between the cellular processes governing longevity and carcinogenesis [33,34]. This observation is even more stark when only considering human somatic solid cancers [35]. When the data for human solid tumors are presented as a function of increasing age, an intriguing phenomenon is observed: a clear inflection point that occurs at just after 50 years of age [36,37] (shown by a circle in Figure 2A). This inflection point is preceded by an initially gradual slope (referred to as the early or E-slope) but is followed by a very steep curve (referred to as the S-slope), indicating a significant increase in human cancer incidence after age 50 (Figure 2A).

These results strongly suggest that some change in, or dysregulation of, critical biological processes and/or cellular reparative pathways occurs at this inflection point, putting us at an increased risk for somatic tumors [34]. However, while it is tempting to suggest this is due to loss of a specific protein family, that seems unlikely. It is more reasonable to assume that the inflection point is due to a large host of proteins and signaling pathways that maintain a cell's homeostatic poise. As such, our laboratory, as well as many others, is interested in the changes that occur in the cell at this critical inflection point that marks the transition to the tumor-permissive phenotype.

This unique and potentially informative inflection point that is observed in human somatic solid tumors is also observed in almost all species, including mice [38,39]. Analysis of this longevity data in multiple species, *C. elegans* being a common example, shows an initial long, flat slope, followed by an inflection point, and finally a steep curve [40-42], similar to the curve observed in humans. While these results clearly suggest that both aging and somatic human tumors share similar curves, the more interesting question is whether there is a correlative and/or mechanistic relationship between these two curves that involves, at least in some part, the biology of sirtuin proteins.

In this regard, several research groups, most notably the Guarente laboratory, suggest that the time to the inflection point of the longevity curve is directed, at least in some part, by the sirtuin protein family. This suggestion is based on the observation that overexpression of the sirtuin proteins in *C. elegans* increased overall lifespan, while in contrast, deletion of these genes resulted in the opposite effect [40-42]. Perhaps the most interesting aspect of these seminal studies is that loss of and/or enforced expression of the *C. elegans* sirtuins altered the length of the early slope while the slope of the curve after the inflection point remained unchanged (Figure 2B). This result suggests two obvious possibilities: (1) the inflection point is, to some degree, directed by sirtuin activity or (2) there may be a threshold of cellular repair directed by sirtuins, and at some point, cellular damage exceeds repair, potentially playing a role in the appearance of the inflection point. However, it is safe to assume that there are likely many more plausible explanations as well as other proteins that direct this cellular process.

### Caloric restriction, mitochondrial energy metabolism, aging, and human carcinogenesis

**Figure 3:**
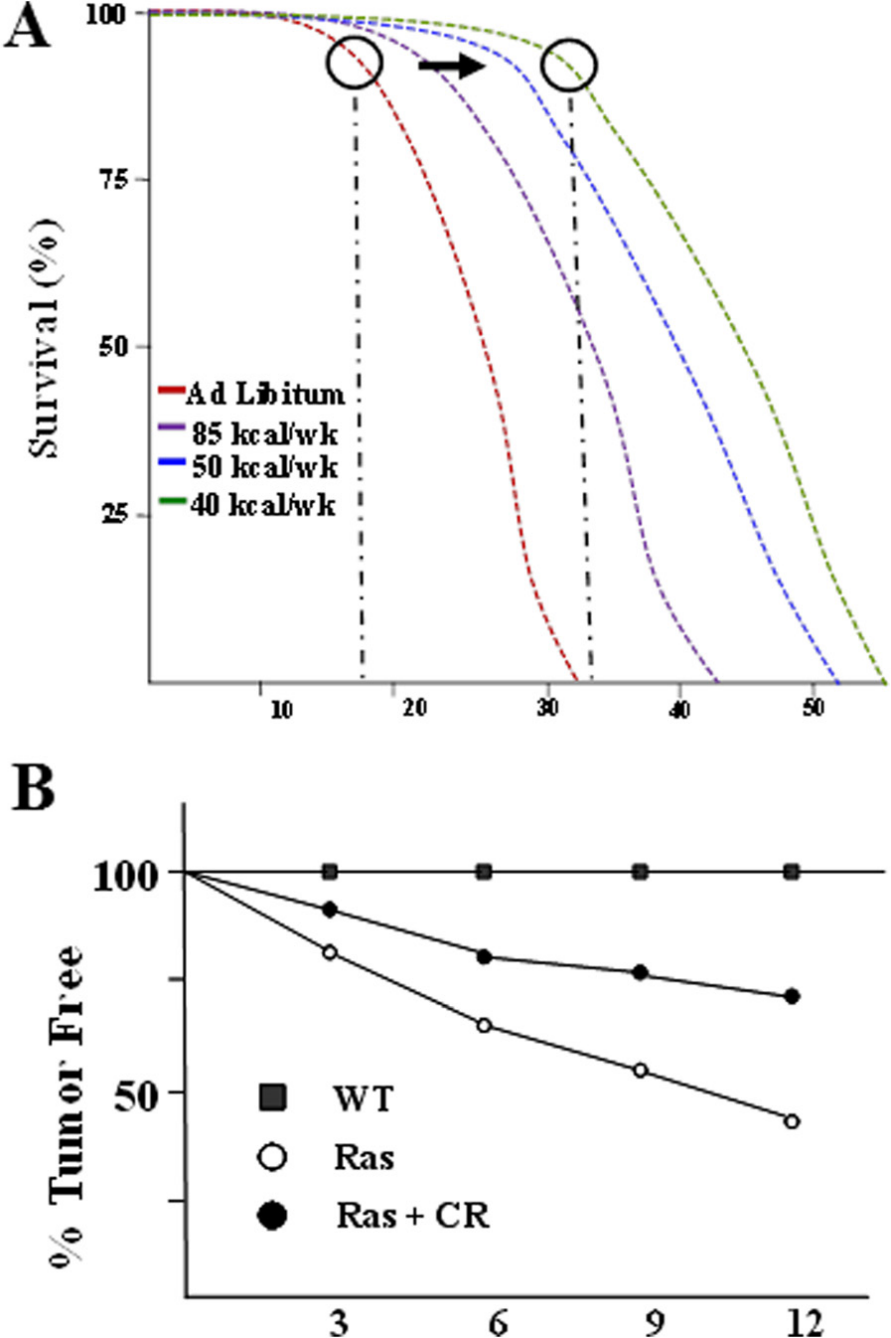
**Effects of caloric restriction on murine survival and carcinogenesis. (A)** Overall survival or longevity in mice on a standard ad libitum diet or CR diets consisting of 85, 50, or 40 kcal/week. The black circles highlight the inflection points of the survival curves on the *ad libitum* and 40 kcal/week diets. **(B)** The incidence of pancreatic cancers in an LSL-KrasG12D genetic knock-in mouse model on either an *ad libitum* diet or a CR diet. Results are presented as %survival or %tumor free, respectively, as a function of mouse age.

It is a well-established observation that animals on a CR diet exhibit significant health-related effects, including an increase in overall lifespan, which is, of course, also dependent upon other nuanced factors [14,43,44]. However, if one carefully analyzes these results, the closer a diet is to the maximum CR level (i.e., 70% of *ad libitum*), the greater the increase in murine longevity (Figure 3A) [45]. In addition, it also appears to some significant extent that the increase in lifespan is due to increasing the length of time to reach the inflection point (Figure 3A) and not the other portions of the longevity curve [45]. Furthermore, it is well established that murine models genetically designed to induce specific types of tumors also exhibit a decrease in spontaneous disease when placed on a CR diet [46], as shown by an example of a murine model of mutant Kras-driven carcinogenesis (Figure 3B). Similarly, a decrease in spontaneous pancreas tumors was also observed in a rat model for pancreas malignancies [47]. While there are no rigorous data in humans that definitively link either an increase in lifespan or decreased incidence of malignancies to CR, there are multiple reports of soft data to suggest this [33,34].

It is also well established that there is a strong relationship between aging and mitochondrial function [48-51], suggesting that the accumulation of mitochondrial damage results in cellular damage that may include that due to reactive oxygen species (ROS), mtDNA damage, etc. as well as a decrease in lifespan. In this regard, three of the seven mammalian sirtuins are found in the mitochondria, including SIRT3 and SIRT4 [52]. These results suggest that the mitochondrial sirtuins (or at least SIRT3 and SIRT4) may respond to changes in cellular and nutrient stress by modification of downstream target proteins [27-31]. While this has not been clearly shown for SIRT4, it has been shown that SIRT3 activity is activated by CR and fasting [29-31].

If sirtuins, including the mitochondrial sirtuins, sense nutrient status (i.e., fasting), it seems reasonable to propose that one function of these proteins is to match energy production to cellular need as well as energy consumption. In addition, it would also suggest that the regulation of the mitochondrial acetylome would play a role, at least in part, in matching cellular energy need to availability [17,53]. Reversible acetylation of lysine is a post-translational modification that neutralizes the positive charge of this amino acid, potentially altering the 3-dimensional structure of a protein as well as its enzymatic function [54,55]. Thus, it has been proposed that at least one function of the sirtuin gene family is the regulation and maintenance of the metabolome via the deacetylation of specific downstream target proteins that direct the specific pathways in the mitochondria that direct energy productions [15,56].

**Figure 4:**
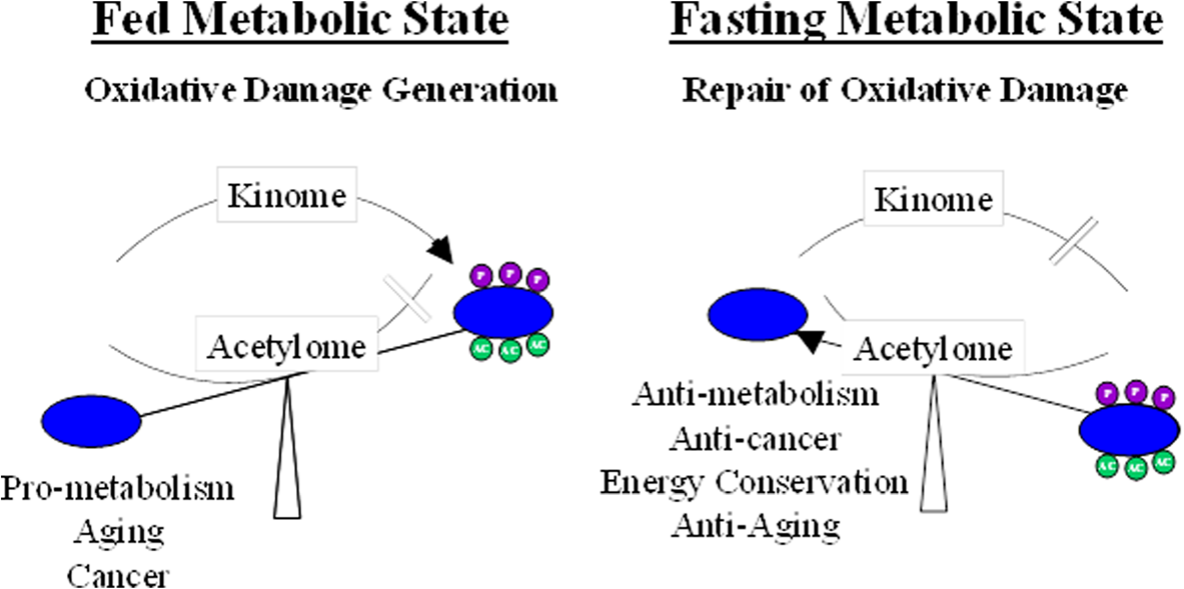
**Schema outlining the opposing effects of the kinome and the acetylome on metabolism in response to energy availability.** Fed conditions favor oxidative damage due to the induction of pro-metabolism pathways that are induced by insulin and other cytokines that signal a high-energy availability status that would inactivate sirtuins. A fasting state is proposed to activate sirtuins and should induce cellular pathways that conserve or increase cellular efficiency, resulting in energy conservation and preservation of cellular integrity.

These results imply that sirtuins in general, and mitochondrial sirtuins specifically, are nutrient status sensing proteins that transmit a signal to downstream target genes, as well as critical mitochondrial processes, via protein deacetylation. We would propose that the mitochondrial sirtuins respond to what has often been described in most physiology courses as the fed versus the fasting organismal/cellular state [57]. In our adaptation of this model, it is proposed that in a fed state, the cells sense that energy packets (i.e., food) are readily abundant and as such, cellular processes are activated. This favors a pro-metabolism, pro-aging, and pro-carcinogenic phenotype. The activation of these processes, potentially induced by insulin secretion from the pancreas, would inactivate sirtuins, resulting in a general cellular state of increased protein acetylation (Figure 4). In addition, insulin would also activate a series of kinases that would activate pro-metabolism, and it is suggested that these pathways would result over time in aging and a carcinogenesis-permissive phenotype. Finally, a pro-metabolism status may drive aging at the organismal levels while different degrees of aging may occur at the cellular and/or tissue/organ level, and it is this combination of forces that results in the more complex signs and symptoms of increasing age on a species.In contrast, when an organism is a fasting state, which was likely quite often for evolutionarily primitive man, it seems reasonable to propose that a metabolic state would be established that maximizes the efficiency of energy generation as well as energy use. In this environment, is seems reasonable to propose that the activity of the mitochondrial sirtuins, as well as perhaps the entire sirtuin family, would be activated, resulting in a generalized deacetylated status (Figure 4). This would result in a cellular phenotype that would be energy conserving, anti-metabolism, anti-aging, as well as anti-carcinogenic. However, this is presented as a very simplistic model that may apply mostly to SIRT3 and the mitochondrial, and it is clear that changes in both acetylation and phosphorylation are much more complex as well as the interplay between this PTM on specific proteins. Finally, does this provide some insight as to why sirtuins might be fidelity or TS proteins? In this regard, it could be proposed that one consequence of making cells adapt to become more efficient is the induction of pathways that might also result in a cellular and/or organellar reparative phenotype.

### Mitochondrial SIRT3 acts as a tumor suppressor

Lysine acetylation appears to be not only important but also perhaps the primary post-translational modification used to adapt cells to periods of fasting and to direct the activity of specific mitochondrial proteins [58-60]. In this regard, several proteomic surveys have identified a disproportionately high number of acetylated proteins in the mitochondria, which contain reversible acetyl-lysines associated with energy homeostasis [23,61]. SIRT3 is the primary mitochondrial deacetylase, and genetic knockout of *Sirt3* alters a significant number of mitochondrial protein acetylation sites [62], including proteins involved in the generation of ATP [26]. Therefore, it seems reasonable to propose that lysine acetylation of mitochondrial proteins may serve to both maintain and regulate overall mitochondrial metabolism and function. Therefore, we believe that it is logical to hypothesize that SIRT3 acts as a metabolic sensing protein to direct the regulation of mitochondrial energy metabolism and ATP synthesis, the detoxification of mitochondrial ROS, and other biological processes essential for proper mitochondrial function. In addition, as discussed above (Figure 4), it is likely that SIRT3 senses decreased nutrient availability and responds by increasing the efficiency of mitochondrial pathways that generate ATP, shifting energy away from glycolysis and towards oxidative phosphorylation.

It is a well-established observation that there is a link between dysregulation of mitochondrial function in cancer cells, which exhibit a significant increase in glycolysis, and aberrant cellular metabolism. This link is commonly referred to as the “Warburg Effect” [26,63]. This has also been observed to be a function of age, suggesting a potential mechanistic link between the cellular processes governing mitochondrial function, longevity, and the development of cancers [34]. Finally, tumor cells also appear to have increased ROS levels that may be due to aberrant metabolism, either through increased production or decreased enzymatic detoxification, either of which may result in oxidative stress and persistent oxidative cell damage, adversely affecting genome stability. Increased ROS levels are considered an early event in carcinogenesis and, under specific cellular conditions, can further affect cell dedifferentiation, tumor initiation, and progression. These results provide strong evidence to support the hypothesis that mitochondrial dysregulation plays a significant role in the multi-hit process of cell transformation and ultimately carcinogenesis. Based on these results, it has been proposed that mitochondrial proteins, such as SIRT3 and SIRT4, may function as critical regulators at the crossroads between metabolism, aging, and aging-related human illnesses such as cancer [64]. Finally, the logical extension of this idea is that the loss of SIRT3 activity, by one of several potential mechanisms, would create a cellular environment permissive for age-related cancers [29].

Therefore, mice lacking the mitochondrial *Sirt3* gene were established to determine whether SIRT3 is a TS protein, in which case, cells or mice lacking Sirt3 would likely display a tumor-permissive phenotype. In this regard, our laboratory has shown that mice lacking *Sirt3* do not exhibit an obvious or early *in vivo* phenotype or other physiological abnormalities; however, the livers of these mice exhibit a significant increase in acetylated mitochondrial proteins, as compared to the wild-type mice [62]. Moreover, when these mice or primary tissue cultures derived from them were treated with various stress factors, such as oxidative stressors, chemical-hormonal, or ionizing radiation, they displayed physiological phenotypes consistent with increasing age, including cardiac hypertrophy [28,65], carcinogenesis [29,66,67], fatty liver [27,68], radiation-induced liver damage [31], and age-related hearing loss [32,64]. Interestingly, a common observation in each of these studies showed loss of *Sirt3*-induced higher steady-state levels of ROS as well as oxidative stress.

While fidelity proteins, whether in the nucleus, cytoplasm, or the mitochondria, appear to have multiple downstream targets, it seems reasonable to suggest that the observed increase in ROS levels in cells deficient in SIRT3 may contribute to the development of age-associated pathologies. Thus, loss of *Sirt3* may induce aberrant mitochondrial metabolism, and when the cells are exposed to additional endogenous and exogenous insults that also result in stress, there may be resultant intracellular redox imbalance that may have deleterious biological effects.

Therefore, there are two questions that must be addressed: (1) how does SIRT3 regulate mitochondrial metabolic homeostasis? (2) What are the downstream targets involved in this regulatory process?

Recent studies have demonstrated that Sirt3 regulates the tricarboxylic acid cycle by deacetylating isocitrate dehydrogenase [32], glutamate dehydrogenase (GDH) [69], and acetyl-CoA synthetase [61,70]. SIRT3 also regulates the fatty acid cycle by deacetylating long-chain acyl-coenzyme A dehydrogenase and 3-hydroxy-3-methylglutaryl coenzyme A synthase 2 [26,60,70]. Our group and others have observed that subunits of the electron transport chain (complexes I–III and ATP synthase) are also the substrates of SIRT3 [71,72]. In addition, SIRT3 deacetylates manganese superoxide dismutase (MnSOD), altering its activity of superoxide removal [31]. Combined, these results strongly suggest that SIRT3 is the primary mitochondrial deacetylase serving to direct mitochondrial energy production as well as to limit the accumulation of mitochondrial ROS.

*In vivo* loss of *Sirt3* in mice exhibit dysregulation of mitochondrial functions including increased mitochondrial DNA damage in the liver, reduced ATP production, an increase in mitochondrial ROS (including superoxide), as well as increased ER/PR positive breast malignancies [29]. Similarly, when *Sirt3* knockout mouse embryonic fibroblasts (MEFs) were challenged with various stress factors, these cells had loss of contact inhibition and were subsequently immortalized/transformed by infection with a single oncogene, suggesting that SIRT3 may function as a TS [29,31].

Interestingly, there is a significant decrease in SIRT3 levels in human tumors compared to normal tissue controls. While these results appear to suggest that SIRT3 is a genomically expressed, mitochondrially localized TS, the mechanism through which SIRT3 protects against tumorigenesis is unclear. As discussed, many substrates of SIRT3 are tightly linked with energy homeostasis and ROS production. Therefore, it has been suggested that increased mitochondrial oxidative stress contributes to human carcinogenesis. Our data support this hypothesis, as murine breast tissue lacking *Sirt3* exhibited an increase in steady-state ROS. Similarly, human breast tissue samples also displayed increased mitochondrial superoxide levels coinciding with decreased *Sirt3* expression. Sirt3^-/-^ mouse hepatocytes [28,29] and cardiomyoctes [28,65] also presented with significantly higher basal superoxide levels, which were observed to further increase when exposed to different types of exogenous cellular stress.

**Figure 5:**
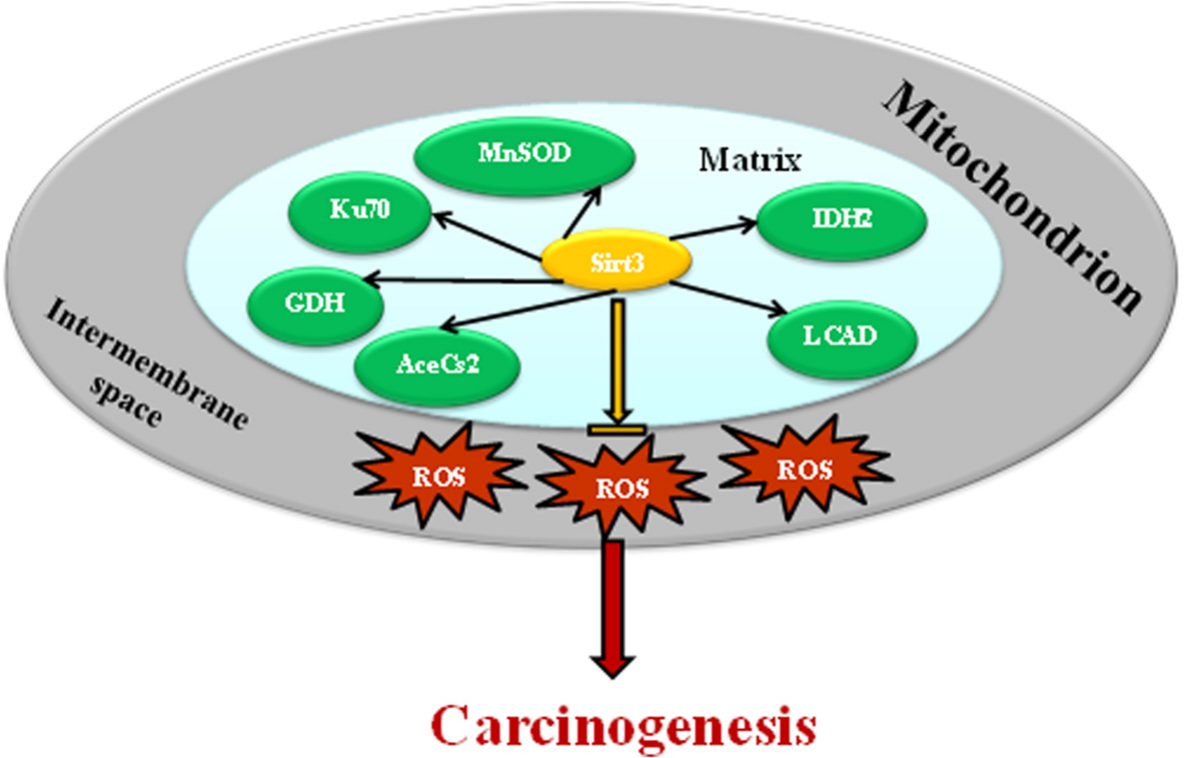
**Schema outlining the multiple mechanisms by which SIRT3 blocks ROS production, thereby preventing carcinogenesis.** Loss of SIRT3 results in mitochondrial dysregulation as well as increased ROS, due in part to increased mitochondrial protein acetylation, including that in MnSOD, and decreased MnSOD detoxification activity as well as other downstream target proteins deacetylated by SIRT3. The increase in ROS is thought to be an early event in the *in vivo* carcinogenesis observed in mice lacking *Sirt3*.

In recent years, three seminal papers have been published that demonstrated that deacetylation of MnSOD by SIRT3 directs its enzymatic activity [30,31,73]. Furthermore, in several tissue culture experiments, co-infection of lenti-MnSOD not only decreased mitochondrial superoxide levels but also prevented immortalization of Sirt3^-/-^ MEFs by a single oncogene [31]. These experiments were confirmed using a MnSOD construct in which lysine 122 was mutated to arginine (MnSOD122K-R), resulting in a constitutively active, dominant positive protein [31]. Co-infection of lenti-MnSOD122K-R also prevented immortalization of Sirt3^-/-^ MEFs by a single oncogene. In contrast, co-infection with dominant negative mutant MnSOD gene (lenti-MnSOD122K-Q) mimicking a constitutively acetylated lysine failed to prevent immortalization by infection with a single oncogene [31]. Finally, it has also been shown that infection with the lenti-MnSOD122K-R gene prevented tissue culture transformation with exogenous agents, including ionizing radiation and stress-induced increases in cellular ROS [31]. These experiments strongly suggest that aberrant mitochondrial superoxide metabolism plays a significant role in the tumor-permissive phenotype (Figure 5) observed in cells lacking *Sirt3*.

Many studies also suggest that changes in the steady state level of ROS may play a critical role in how the mitochondria communicate with other parts of the cell and further lead to changes in gene expression, cell proliferation, and apoptosis [29,74-78]. In support of this hypothesis, Venkataraman et al. showed that overexpression of MnSOD in PC-3 cells resulted in a delay of the G1-S phase transition. This delay was mediated in part by modulation of the redox status of the cell through the increased levels of H_2_O_2_[79]. In addition, Karawajew et al. demonstrated that mitochondrial ROS serve as second messengers by guiding p53 translocation to the mitochondria, leading to the activation of apoptosis and p53 target gene expression [75]. They also showed that treatment of cells with oligomycin, an inhibitor of ATP synthase, prevents stress-induced mitochondrial accumulation of p53 and abrogates p53-dependent apoptosis by reducing mitochondrial ROS levels [75]. These results strongly support the hypothesis that the alteration of mitochondrial ROS production, via changes in MnSOD enzymatic activity (Figure 5) or mitochondrial metabolic homeostasis, represents a potential mechanism for inter-compartmental cellular communication and may play a role in SIRT3 deficiency-induced aging-related cancers.

Finally, several studies also indicate that the acetylation statuses of SIRT3 substrates like acetyl-CoA synthetase, GDH, long-chain acyl-CoA dehydrogenase (LCAD), succinate dehydrogenase, and mitochondrial ribosome subunit MRPL10 are frequently altered in human cancers. Interestingly, SIRT3 has also been shown to have pro-apoptotic or anti-apoptotic effects on different cell types, and at least one mechanism involves deacetylating Ku70, preventing the release of BAX into mitochondria [80]. Although the detailed mechanism of the connection between these mitochondrial protein acetylation and carcinogenesis events is still unclear, these results provide evidence that the mitochondrial acetylome may play an important role in the cellular damage and tumor-permissive phenotype (Figure 5).

### SIRT4 functions as tumor suppressor by directing glutamine metabolism

**Figure 6:**
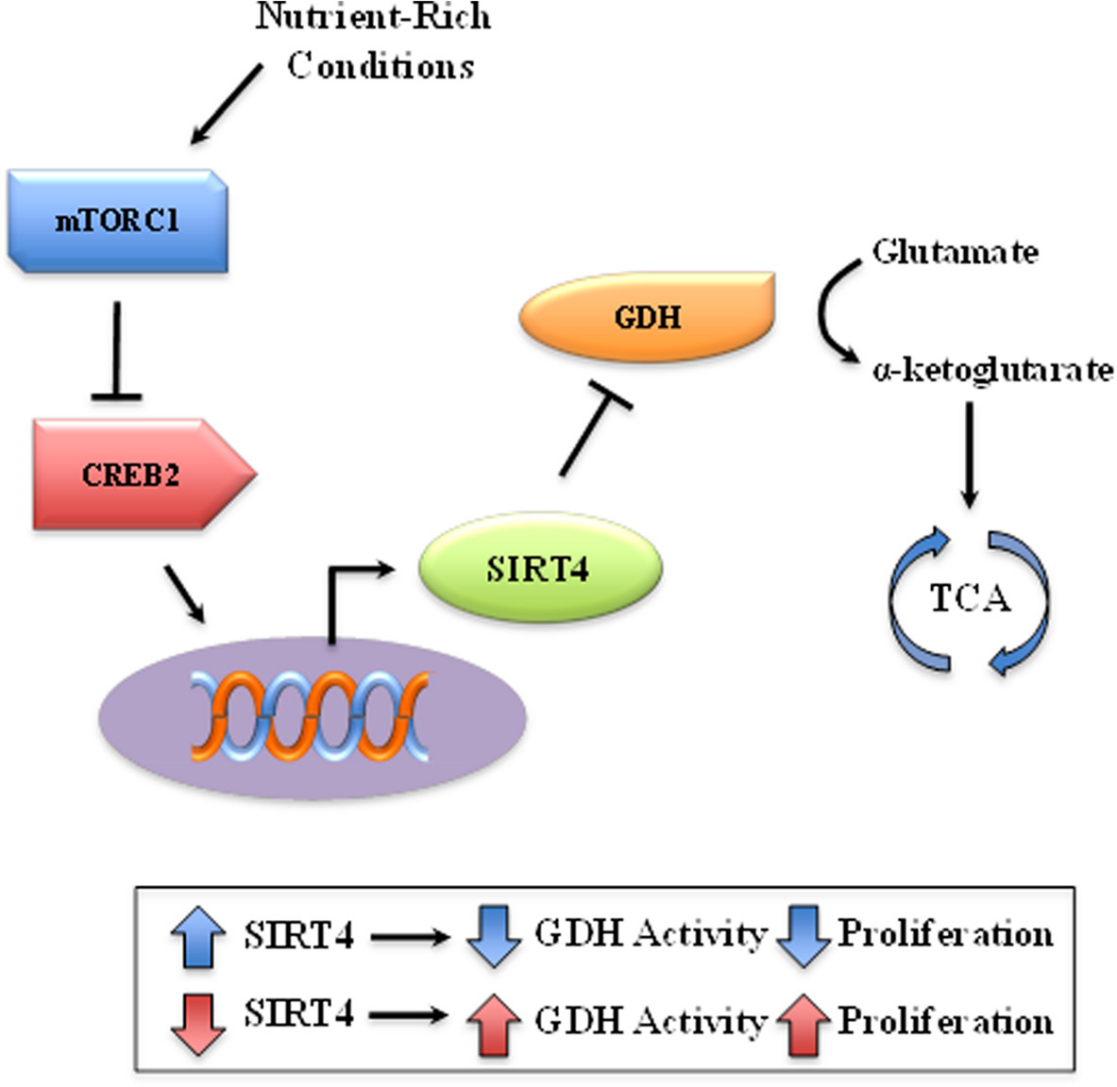
**Schema outlining the proposed pathway by which SIRT4 regulates proliferation.** It is proposed that under nutrient rich conditions mTORC1 inhibits CREB2, decreasing the expression of SIRT4. When SIRT4 activity is decreased, which is observed in the *Sirt4* knockout mice, and what might be expected with increasing age, the glutamate/αketoglutarate and TCA cycles are dysregulated. As such, it is suggested that this plays a role, at least in some part, in the tumor-permissive phenotype in mice lacking *Sirt4*.

Recent evidence suggests that SIRT4 may also have a role in cell metabolism and carcinogenesis. Like SIRT3, SIRT4 regulates metabolic function through variety of mechanisms. While SIRT3 directs post-translational modifications via protein deacetylation, SIRT4 affects its targets largely through NAD-dependent ADP-ribosylation (Figure 6) [20]. SIRT4 is expressed in several cell types including liver, kidney, testis, striated muscle, and vascular smooth muscle as well as the insulin-producing β cells in the islets of Langerhans [81].

SIRT4 is activated in response to genotoxic stress and is required for the block in glutamate metabolism allowing for a proper DNA damage response [82]. While SIRT4 exhibits no deacetylase activity on histones or serum albumin [81], recent findings suggest SIRT4 deacetylates malonyl CoA decarboxylase (MCD) under low nutrient conditions. MCD produces acetyl CoA from malonyl CoA, the latter providing a carbon skeleton for lipogenesis under nutrient-rich conditions [83]. When deacetylated by SIRT4, MCD functions less efficiently, and animals lacking SIRT4 present with increased MCD activity, dysregulated lipid metabolism, and protection against diet-induced obesity (Figure 6). Therefore, SIRT4 opposes fatty acid oxidation, promoting lipid anabolism by regulating MCD function/malonyl CoA levels [83]. Similarly, in both myocytes and hepatocytes, loss of SIRT4 increased fatty acid oxidation gene expression and cell respiration [84].

In pancreas β cell mitochrondria, SIRT4 serves to ADP-ribosylate GDH, a mitochondrial enzyme that converts glutamate to α-ketoglutarate, the activity of which is also modulated by ADP-ribosylation [85]. GDH promotes glutamine/glutamate metabolism, facilitating ATP production and insulin secretion. Once ADP-ribosylated, the enzymatic function of GDH is repressed, leading to \reduced ATP synthesis and less effective insulin secretion in response to exogenous amino acids [86,87].

Clinically, *SIRT4* mRNA expression is reduced in several malignancies, including breast, colon, bladder, gastric, ovarian, and thyroid cancers, though *SIRT4* loss was particularly pronounced in lung cancer patients (Figure 6). Accordingly, mice with whole body knockout of *Sirt4* present with a variety of solid tumors, though most frequently lung tumors [86,88]. In addition, loss of *SIRT4* corresponds with increased aggressiveness in women with breast cancer. Furthermore, overexpression of SIRT4 opposes cell proliferation, transformation, and tumor progression as shown in an *in vivo* murine model [89]. Similarly, loss of *SIRT4* accelerates Myc-induced B cell lymphomagenesis in mice lacking *Sirt4*, and *SIRT4* overexpression sensitizes cells to apoptosis induced by glycolysis inhibitors [88].

Combined, these observations strongly suggest that SIRT4 has tumor-suppressive effects and that its downregulation may serve to facilitate the progression of several human cancers. Loss of SIRT4 appears to be a result of mammalian target of rapamycin complex 1 (mTORC1), a complex consisting of mTOR, Raptor, and mLST8 that is dysregulated in human cancers and activated under nutrient-rich conditions [90]. mTORC1 leads to proteasome-mediated destabilization of cAMP-responsive element binding 2 (CREB2), a key transcriptional regulator of SIRT4. By destabilizing CREB2, mTORC1 reduces *SIRT4* expression, thereby increasing GDH activity and glutamine/glutamate metabolism [89].

## Conclusions

The results discussed above suggest that loss of a single mitochondrial protein leads to the aberrant regulation of the mitochondrial acetylome signaling network that responds to metabolic demands and deacetylates downstream target proteins, resulting in a phenotype permissive for human illnesses associated with aging. In this regard, it is proposed that SIRT3 and SIRT4 respond to changes in cellular nutrient status to alter the enzymatic activity of specific downstream targets to maintain energy production that matches energy availability and ATP consumption. As such, it is proposed that loss of function or genetic deletion of these mitochondrial genes results in a mismatch of mitochondrial energy metabolism, culminating in a cell phenotype permissive for transformation and tumorigenesis. As such, we believe that the *Sirt3* and *Sirt4* knockout mice represent a new paradigm that mechanistically links mitochondrial metabolism, the acetylome post-translational signaling network, and age-related disease including carcinogenesis.

## Abbreviations

CR: calorie restriction; CREB2: cAMP-responsive element binding 2; GDH: glutamate dehydrogenase; HDAC: histone deacetylases; LCAD: long-chain acyl-CoA dehydrogenase; MCD: malonyl CoA decarboxylase; MEF: mouse embryonic fibroblast; MnSOD: manganese superoxide dismutase; mTORC1: mammalian target of rapamycin complex 1; NAD^+^: nicotinamide adenine dinucleotide; ROS: reactive oxygen species; SIRT: sirtuin; TS: tumor suppressors.

## Competing interests

The authors declare that they have no competing interests.

## Authors' contributions

YZ reviewed the Sirt3 studies, participated in the preparation of the figures, and drafted the manuscript. YY participated in manuscript design and coordination and helped to edit the manuscript. DP reviewed the Sirt4 studies, participated in the preparation of the figures, and drafted the manuscript. XZ and AV participated in the design of the manuscript and edited the manuscript. DG conceived of the study and participated in its design and coordination and helped to draft the manuscript. All authors read and approved the final manuscript.
